# Supplementary material for: Structure of the peroxisomal Pex1/Pex6 ATPase complex bound to a substrate
Source: Nat Commun. 2023 Sep 23;14:5942. doi: 10.1038/s41467-023-41640-9 (PMC10518020; doi:10.1038/s41467-023-41640-9)
Supplement: Supplementary file 1 — Supplementary Information [file 41467_2023_41640_MOESM1_ESM.pdf]

# Supplementary Information

# Structure of the peroxisomal Pex1/Pex6 ATPase complex bound to a substrate

Maximilian Rüttermann[1,2], Michelle Koci[3], Pascal Lill[1,2,3], Ermis Dionysios Geladas[1,2], Farnusch Kaschani[4], Björn Udo Klink[1,2], Ralf Erdmann[5], Christos Gatsogiannis[1,2,3,*]

[1]Institute for Medical Physics and Biophysics, University Münster, 48149 Münster, Germany.

[2]Center for Soft Nanoscience (SoN), University Münster, 48149 Münster, Germany.

[3]Department of Structural Biochemistry, Max Planck Institute of Molecular Physiology, 44227, Dortmund, Germany.

[4]Analytics Core Facility Essen, Center of Medical Biotechnology (ZMB), Faculty of Biology, University of Duisburg-Essen, Universitätsstr. 2, 45141 Essen, Germany

[5]Institute for Biochemistry and Pathobiochemistry, Department of Systems Biochemistry, Ruhr-University Bochum, 44780 Bochum, Germany

*To whom correspondence should be addressed.

E-mail: christos.gatsogiannis@uni-muenster.de

**Supplementary Table 1:** Phenix density modification command prompts.

| | Class 3<br>Single-'seam' conformation | Class 4<br>Twin-'seam' conformation |
|---|---|---|
| Local anisotropic half-map sharpening | phenix.local_aniso_sharpen run_half1_class001_unfil.mrc run_half2_class001_unfil.mrc resolution=4.14 local_sharpen=True sharpened_map_file_1=.\Output_command\half1_local_sharp.ccp4 sharpened_map_file_2=.\Output_command\half2_local_sharp.ccp4 box_size_grid_units=384 sharpened_map_file=.\Output_command\full_local_sharp.ccp4 | phenix.local_aniso_sharpen run_half1_class001_unfil.mrc run_half2_class001_unfil.mrc resolution=4.7 local_sharpen=True sharpened_map_file_1=.\Output_command\half1_local_sharp.ccp4 sharpened_map_file_2=.\Output_command\half2_local_sharp.ccp4 box_size_grid_units=384 sharpened_map_file=.\Output_command\full_local_sharp.ccp4 |
| Density modification | phenix.resolve_cryo_em half1_local_sharp_aniso.ccp4 half2_local_sharp_aniso.ccp4 seq_file=.\Input\Pex1_Pex6_yeast.fasta resolution=4.14 blur_by_resolution=True blur_by_resolution_factor=20 box_cushion=15 b_iso=90 dm_resolution=3.6 | phenix.resolve_cryo_em half1_local_sharp.ccp4 half2_local_sharp.ccp4 seq_file=.\Input\Pex1_Pex6_yeast.fasta resolution=4.14 blur_by_resolution=True blur_by_resolution_factor=20 box_cushion=15 b_iso=90 dm_resolution=3.8 |

**Supplementary Table 2:** Cryo-EM data collection and refinement statistics

| Data collection | | |
|---|---|---|
| Microscope | Titan Krios with Cs-corrector and XFEG electron source | |
| Voltage (kV) | 300 | |
| Nominal magnification | 105,000 x | |
| Electron Dose ($e^{-}$/ $Å^2$) | 60 | |
| Number of frames | 60 | |
| Detector | Gatan K3 | |
| Pixel size (Å) | 0.34 (super resolution mode) | |
| Defocus range (µm) | -0.6 to -2.4 | |
| Micrographs | 16,763 | |
| **Reconstruction** | | |
| Software | SPHIRE 1.4, RELION 3.1.4, Phenix 1.20, Cryosparc 4 | |
| Total extracted particles | 1,259,079 | |
| Structure<br>Pex1/Pex6E832Q | Class 3<br>Single-'seam' conformation | Class 4<br>Twin-'seam' conformation |
| Particles | 164,610 | 128,983 |
| Symmetry | C1 | C1 |
| Final average resolution, gold standard FSC=0.143 | 4.1 Å | 4.7 Å |
| Final average resolution after density modification FSC=0.5 | 3.9 Å | 4.3 Å |
| **Model refinement** | | |
| Peptide chains | 7 | 7 |
| Residues | 5564 | 5564 |
| Ligands | ATP: 10, ADP: 2, Mg2+: 10 | ATP: 10, ADP: 2, Mg2+: 10 |
| RMSD Bond length (Å) | 0.003 | 0.004 |
| RMSD Bond angles (°) | 0.751 | 0.881 |
| Ramachandran outliers (%) | 0.04 | 0.00 |
| Ramachandran allowed (%) | 7.26 | 9.8 |
| Ramachandran favored (%) | 92.68 | 90.2 |
| Rotamer outliers (%) | 0.06 | 0.00 |
| MolProbity score | 1.81 | 1.89 |
| Clash score | 6.27 | 6.28 |
| EMRinger score | 1.53 | 0.99 |
| B-factors mean (Protein) | 132.26 | 131.73 |
| B-factors mean (Ligand) | 92.38 | 89.13 |

## Supplementary Figures

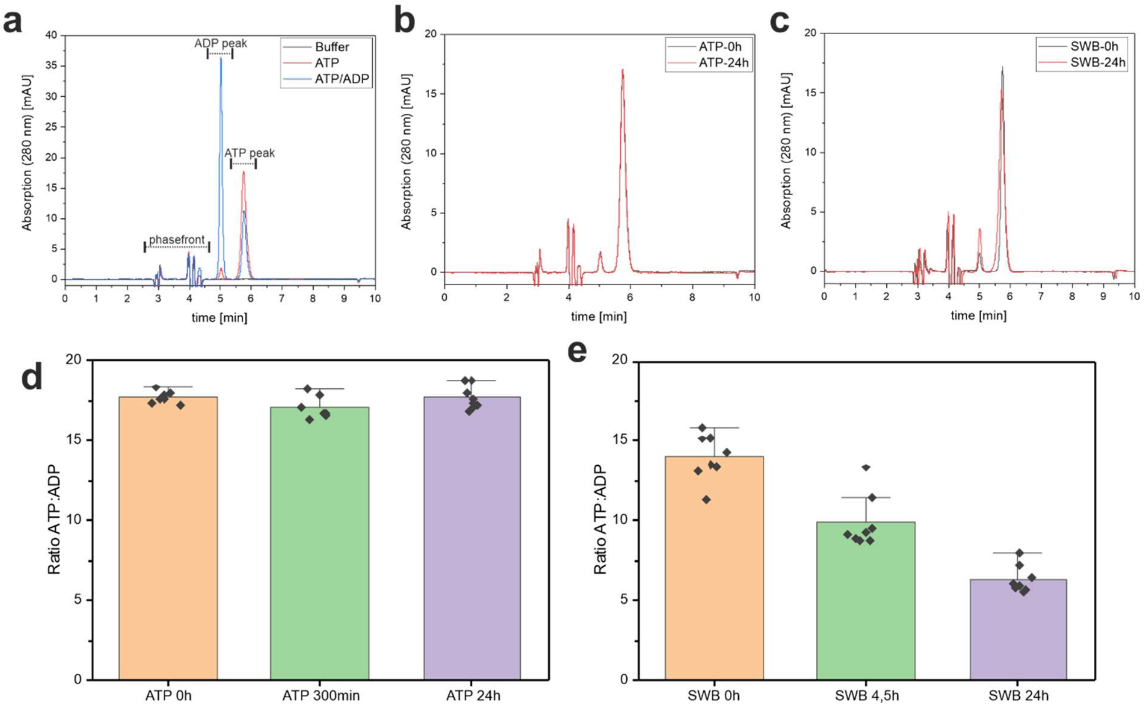


**Supplementary Figure 1: Time-dependent decrease in ATP/ ADP ratio analyzed by HPLC.** Representative chromatograms are shown **(a-c)**. Control experiments was performed with ATP alone. The ATP/ADP ratios in the presence of Pex1/Pex6_WB were obtained by quantification of the results from HPLC analyses at different timepoints upon addition of ATP to the protein sample **(d-e)**. Source data are provided as a Source Data file.

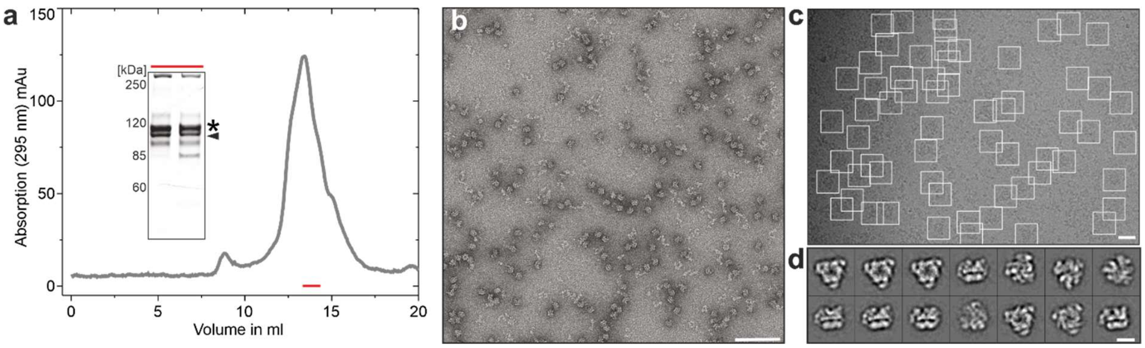


**Supplementary Figure 2: Expression and purification of recombinant Pex1/Pex6_WB. a)** Size exclusion chromatogram (SEC), showing one major peak corresponding to a complex of Pex1 (star) and Pex6_WB (arrow), as confirmed by SDS-PAGE (inset). The red bar indicates fractions, which were pooled for subsequent cryo-EM studies. **b)** Representative micrograph of negatively stained Pex1/Pex6_WB complex after SEC. Scalebar 100 nm. **c)** Representative cryo-EM micrograph of Pex1/Pex6_WB low-pass filtered to 5 Å. Scale bar 20 nm. Boxes represent particles identified by crYOLO. **d)** Representative reference-free 2D class averages of Pex1/Pex6 Pex6_WB complex. Scale bar 10nm.

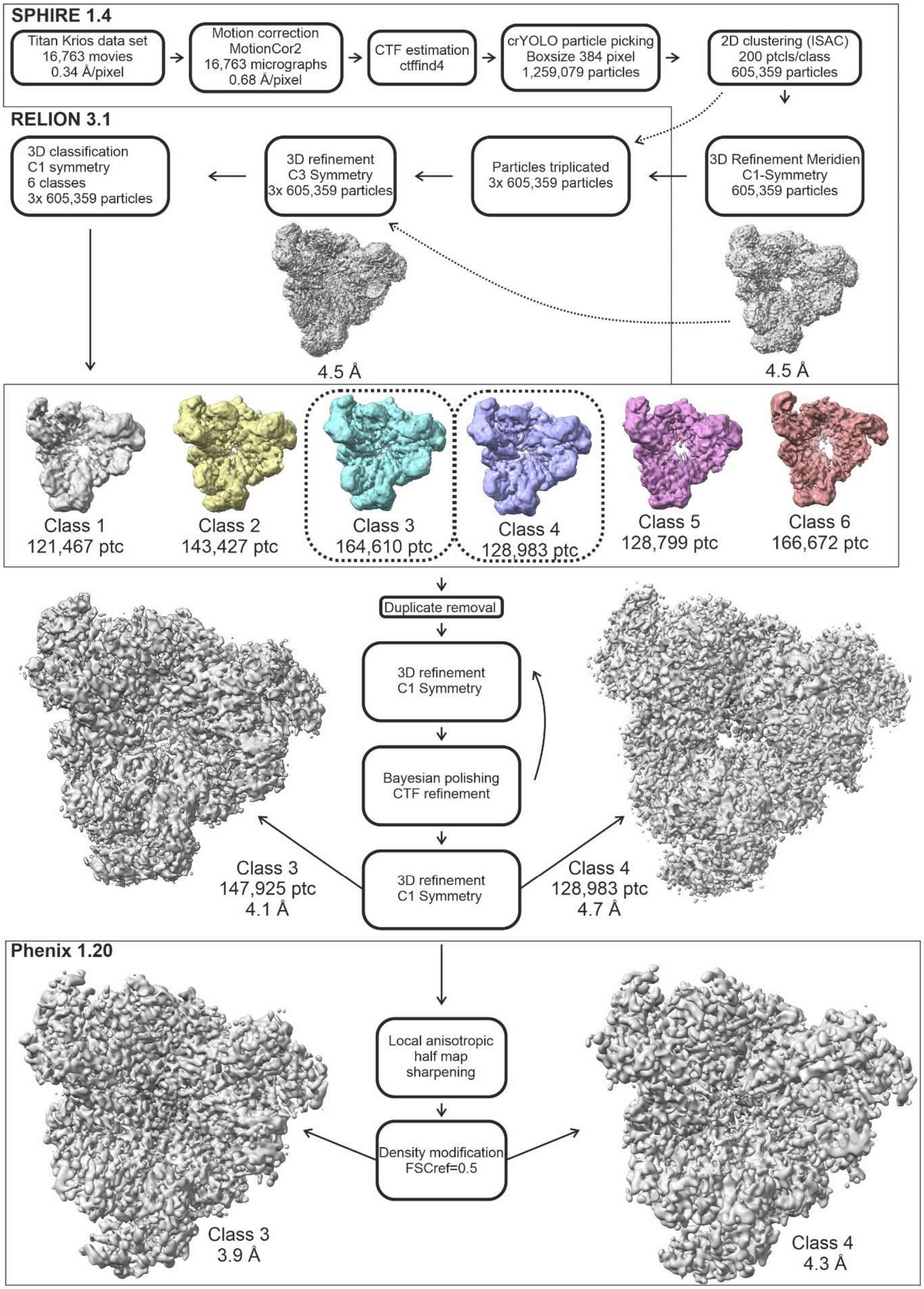


**Supplementary Figure 3: Single-particle cryo-EM processing workflow.** The final maps of class 3 and class 4 from RELION and Phenix have been used for model building. Electron density maps are shown at (class3, single-seam state) 4.42 sigma (2.26 sigma for density-modified) and at (class4, twin-seam state) 4.72 sigma (2.11 sigma for density-modified), respectively.

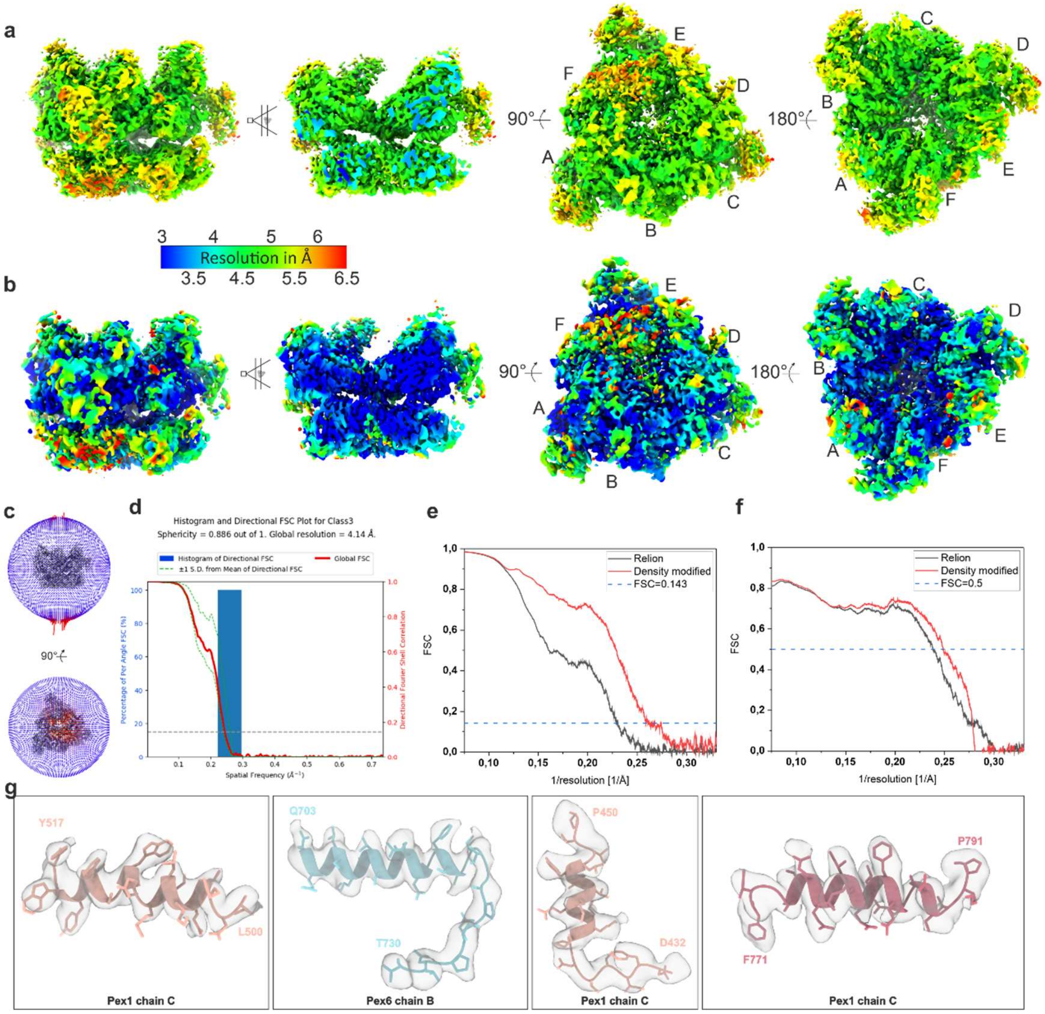


**Supplementary Figure 4: Cryo-EM structure of the single 'seam' state (class 3). a-b)** Different surface views and cross-section of the cryo-EM density map colored according to the local resolution, upon post-processing in RELION at 4.42 sigma (a) and after local anisotropic half map sharpening and density modification using Phenix at 2.26 sigma (b). **c)** Angular distribution for the final round of the refinement. **d)** 3D FSC curve. **e)** Half-map FSC curves and **f)** model-map FSC curves before (gray) and after (red) density modification using phenix. **g)** Representative areas of the density map superimposed with the molecular model. Density-modified map is shown at 2.26 sigma.

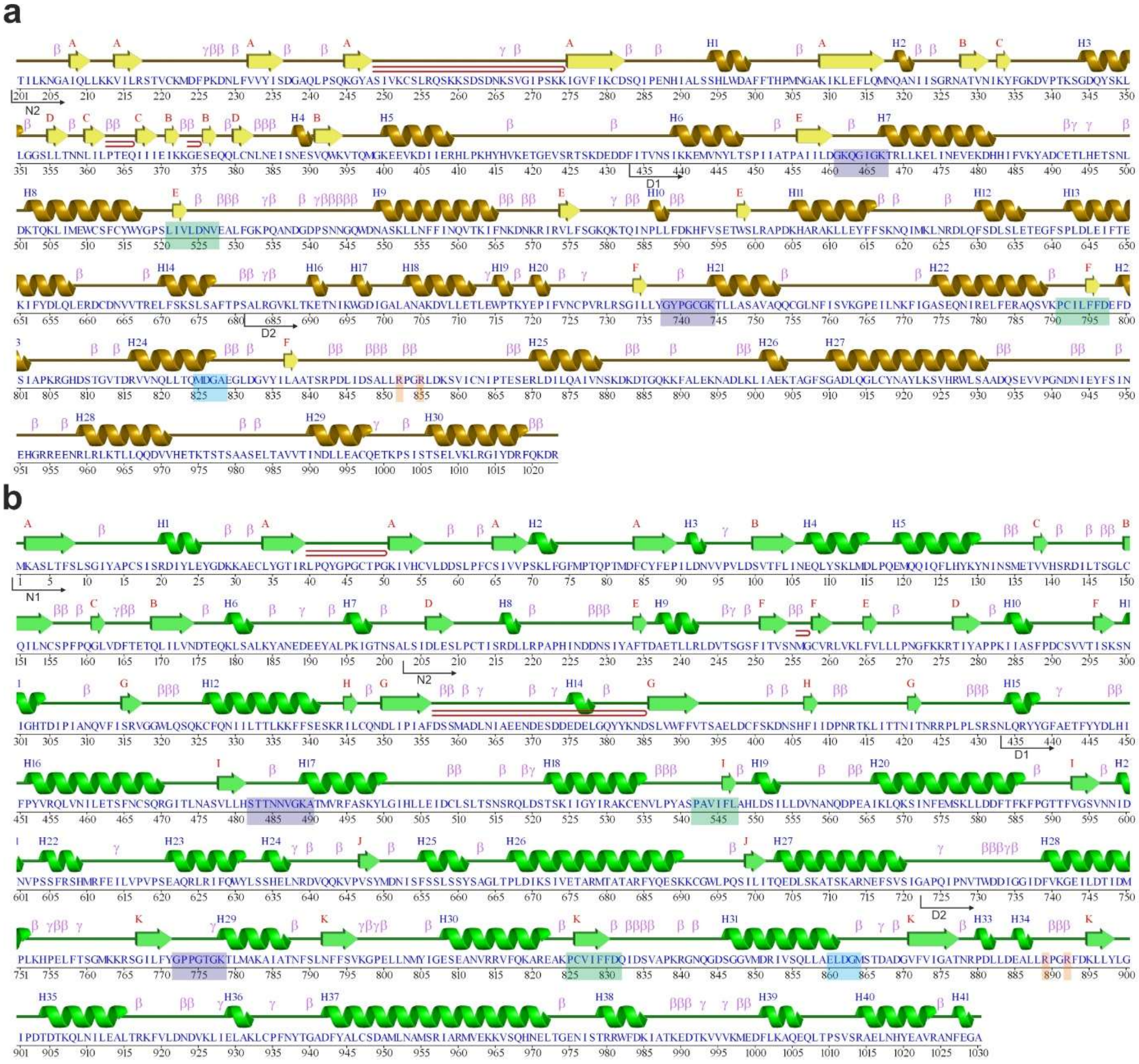

**Supplementary Figure 5. Secondary structure of Pex1(chain c) (a) and Pex6(chain d) (b)** computed with PDBsum (http://www.ebi.ac.uk/pdbsum/)[1]. α-helices are labeled H1, H2, …,H41 and β-strands by their sheets as A, B, …,K. Structural motifs β-turns, γ-turns, and β-hairpins are marked as β, γ, and ⊃, respectively. Important conserved motifs are indicated: Walker A (dark purple), Walker B (green), ISS (cyan), pore loops (red loop) and arginine fingers (orange).

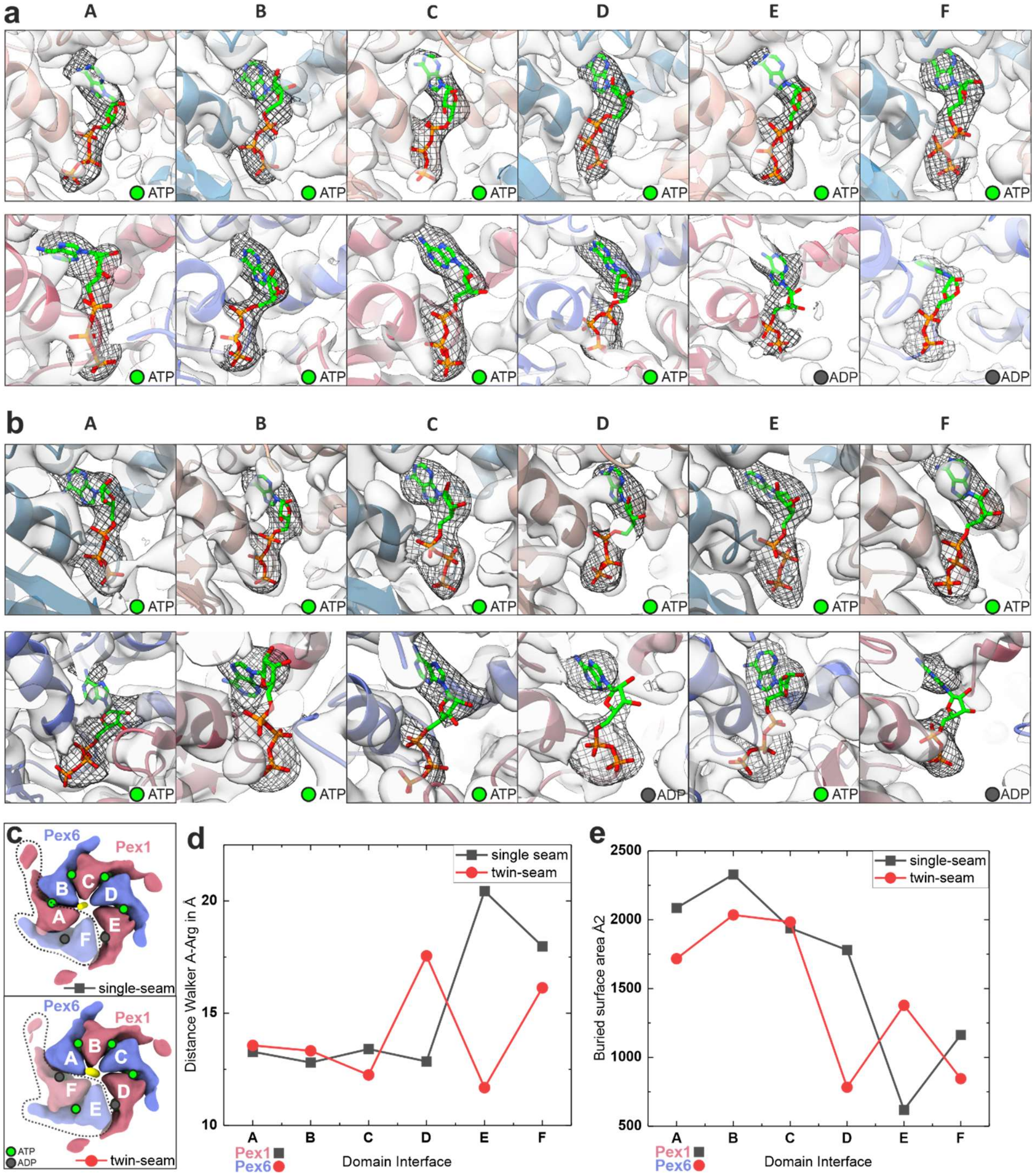


**Supplementary Figure 6: Analysis of the nucleotide binding pockets a-b)** Nucleotide models and corresponding cryo-EM density of the D1 (upper panels) and D2 (lower panels) ring of the single-seam **(a)** and twin-seam **(b)** state. Density-modified map is shown at 2.26 sigma (class3) and 2.11 sigma (class4). A-F indicate Pex subunits. **c)** Position of nucleotide binding pockets and D2-interfaces respective to the disengaged seam (F) domain for the single-seam (upper-inset) and twin-seam state (lower-inset). Letters (from A-F) define the individual subunit position within the stair-case. Domain A occupies the highest position of the spiral. **d)** Measurements of the opening of the nucleotide binding pocket for the single- (gray) and twin-seam (red) state. Shown are the distances between the Cα of Walker A Thr and the Cα of the Arg-finger of the neighboring subunit (Pex1-Pex6 interface: Pex1(T745)-Pex6 (R892); Pex6-Pex1 interface: Pex6(T779)-Pex1(R855). **e)** Contact area between the large and small AAA+ domains of neighboring D2 domains. The buried surface area was measured between the D2 domains of Pex1 (residues 681-1020) and Pex6 (residues 723-1030).

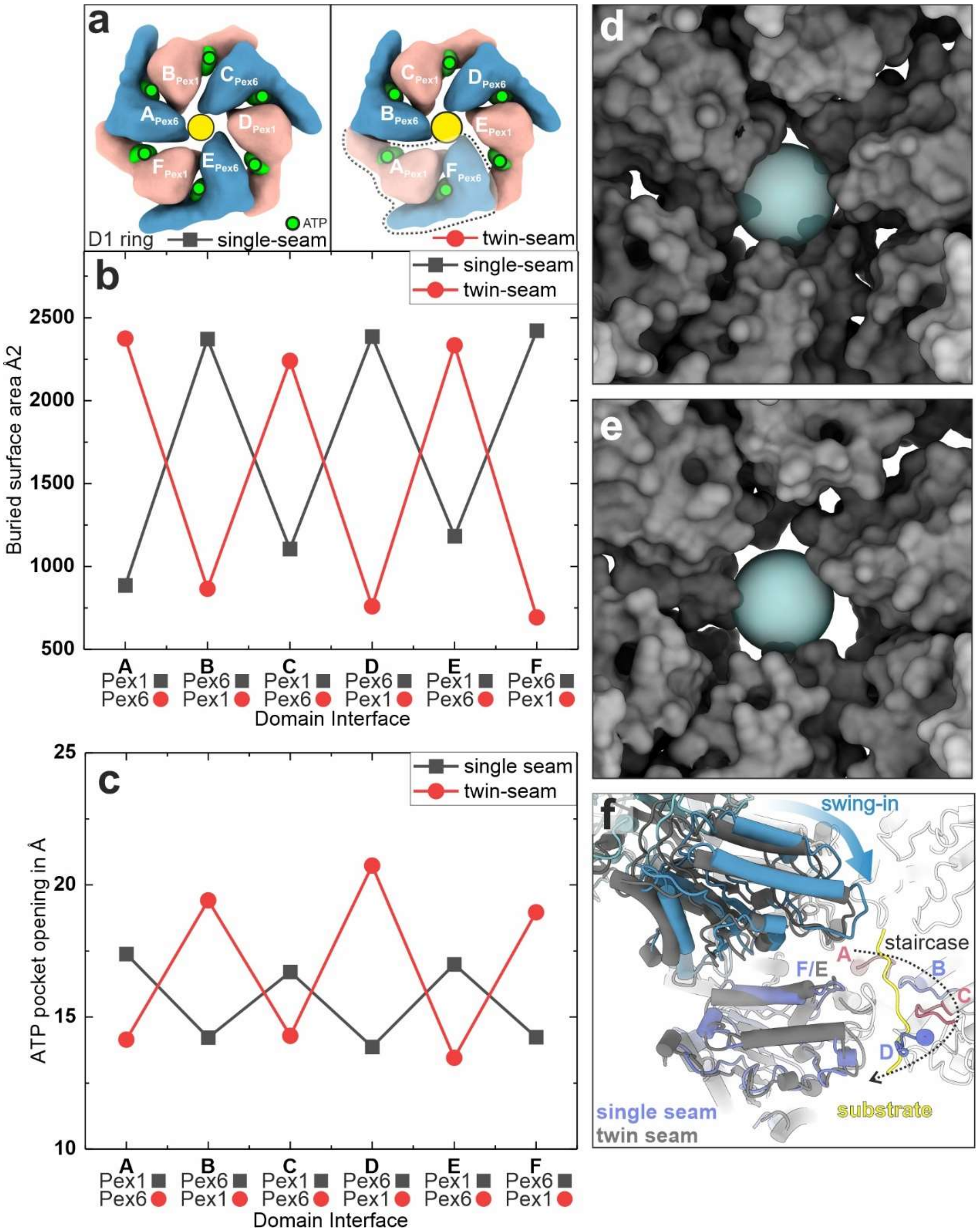


**Supplementary Figure 7: Structural dynamics of the D1 Ring between single- and twin-seam state. a)** Position of nucleotide binding pockets and D2-interfaces for the single-seam (left inset) and twin-seam state (right inset). **b-c)** Shown are measurements of the opening of the b) nucleotide binding pocket (Pex1 T468 to Pex6 D582 and Pex6 T491 to Pex1 K563) and the c) buried surface between adjacent subunits for the single- (gray) and twin-seam (red) state. The buried surface area was measured between the D2 domains of Pex1 (residues 201-680) and Pex6 (residues 201-722). **d-e)** D1 pore of d) single seam state and e) twin-seam state as surface representation. The blue transparent sphere has a diameter of 15 Å. Note the widening of the pore from single- to twin seam-state. **f)** Single-seam state (gray) superimposed on twin-seam state (colored) with chains D (except pore loop) removed to show the interior. Note that the twin-seam (left protomers, gray) swing in on the opposite side of the pore loops 1 staircase, during transition to single-seam (left protomer, colored).

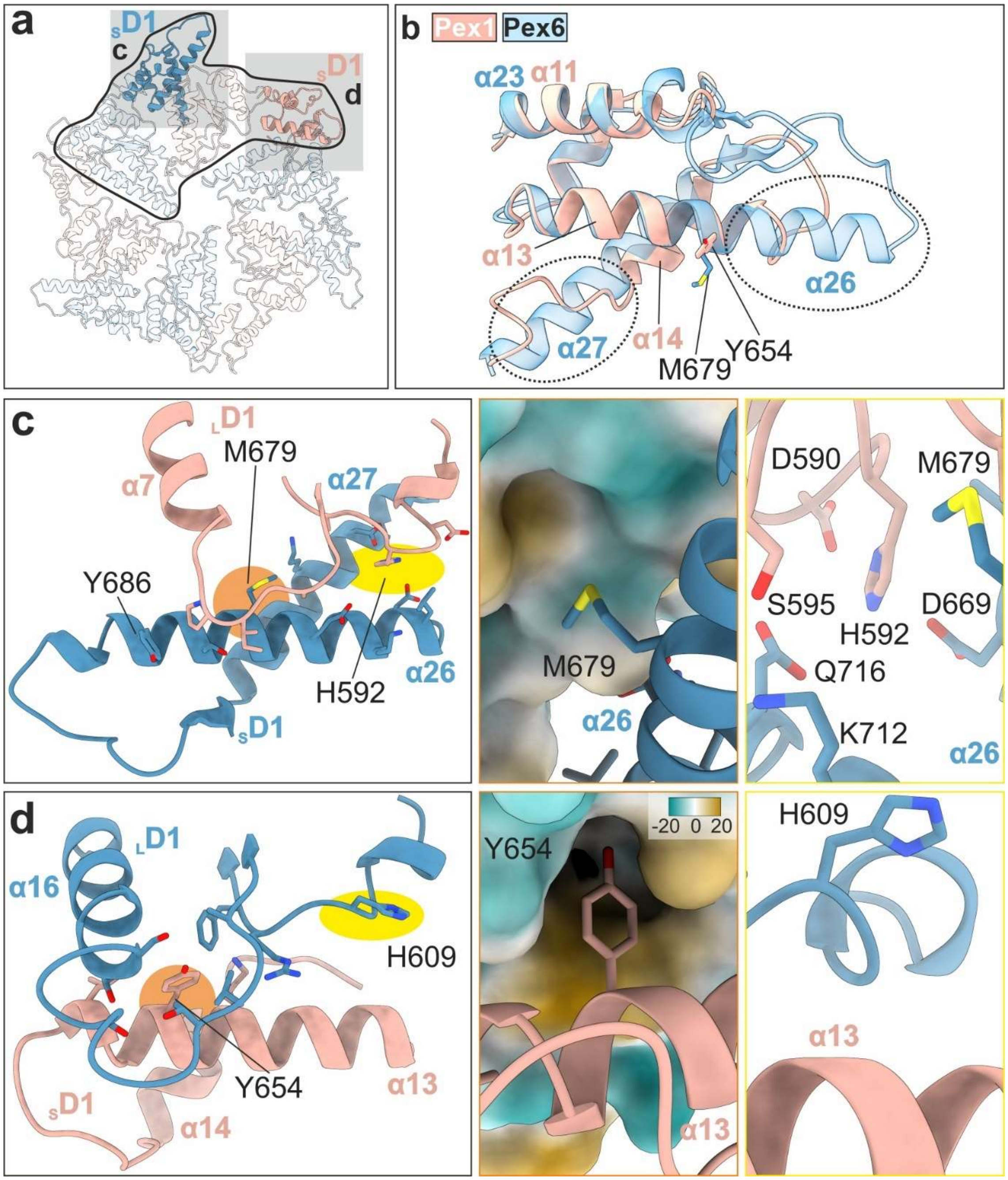


**Supplementary Figure 8: Distinct features of the small D1-ATPase domains of Pex1 and Pex6 determine the "trimer-of-dimers" arrangement in the D1 ring. a)** Top view of the Pex1/Pex6 D1 ring. The Pex6/Pex1 dimer indicated by a black line. The D1 small ATPase subdomains ($_S$D1) of Pex1 (beige) and Pex6 (blue) are highlighted. **b)** Structural alignment of Pex1($_S$D1) (residues 605-685, beige) and Pex6($_S$D1) (residues 621-721, blue transparent). Note the deletion in helix α13 and α14 of Pex1($_S$D1). **c)** Contact site of Pex6($_S$D1) with Pex1($_L$D1) (compact Interface within the dimer). M679 of the prolonged α26 helix of Pex6($_S$D1) (orange highlight) binds to a hydrophobic pocket formed by helix α7, and the loop upstream (residues 447-456) of Pex1($_L$D1). The middle panel shows a close-up view of this interface, with the surface of the pocket colored by hydrophobicity. The conserved histidine H592 of Pex1($_L$D1) (yellow highlight) binds into a negatively charged pocket formed by the prolonged helices α26 and α27 of Pex6($_S$D1). A close-up view of this interface is shown in the right panel.

**d)** Contact site of Pex1($_S$D1) with Pex6($_L$D1) (less compact interface between dimers). Due to the deletion in helix α13 of Pex1($_S$D1), Y654 (orange highlight) binds to the hydrophobic pocket formed by helix α16 and the loop upstream (residues 464-478) of Pex6($_L$D1). The middle panel shows a close-up view of this interface, with the surface of the pocket colored by hydrophobicity. Interestingly, due to the deletion, the analogous conserved H609 of Pex6 ($_L$D1) (yellow highlight, right panel) does not contact Pex1($_S$D1).

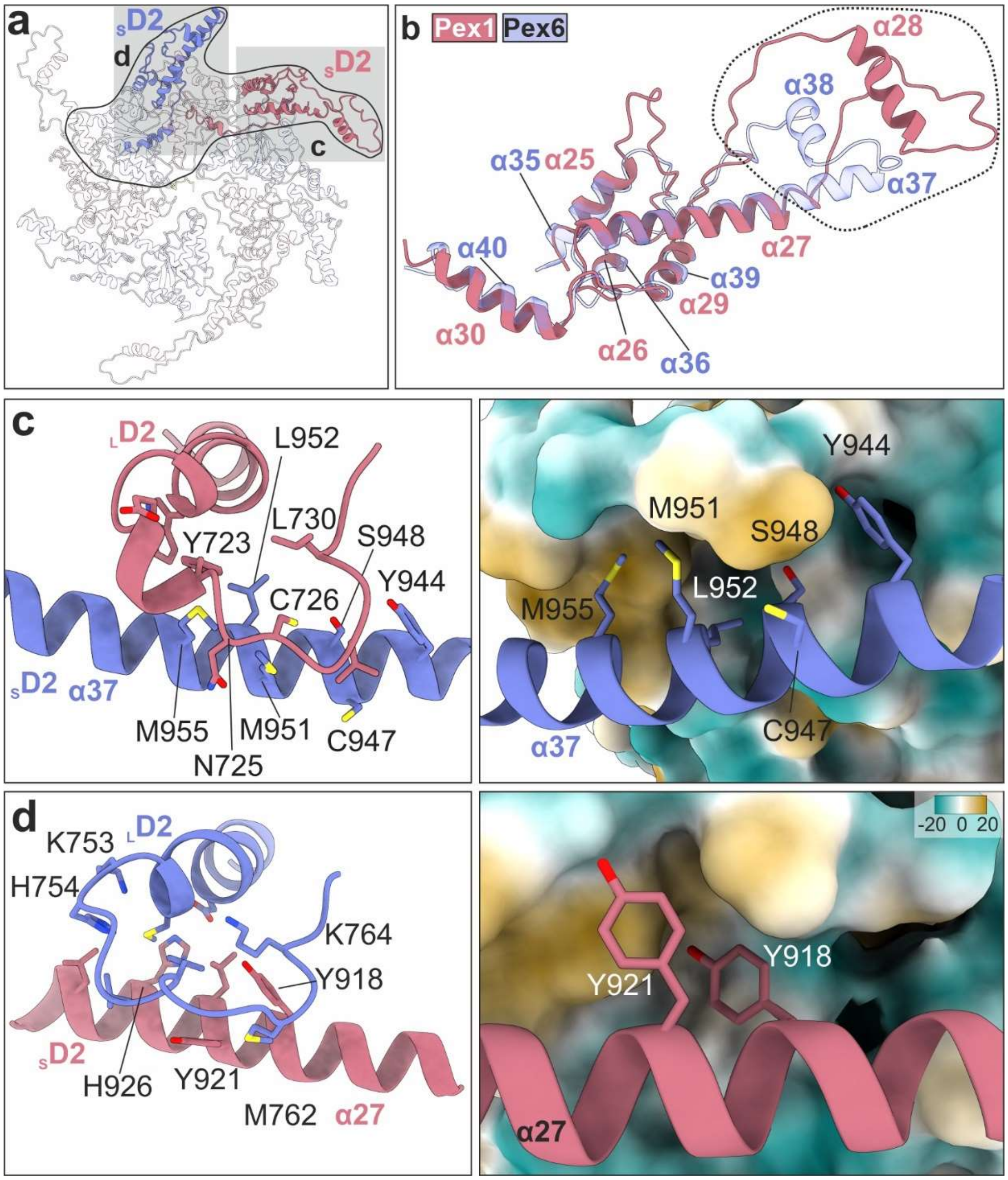


**Supplementary Figure 9: Distinct features of the small D2-ATPase domains fine-tune trimer-of-dimer arrangement in the D2-ring. a)** Top view of the Pex1/Pex6 D2 ring. The Pex6/Pex1 dimer is indicated by a black line. The D2 small ATPase subdomains ($_S$D2) and large ATPase domains ($_L$D2) of Pex1 (red) and Pex6 (blue) are highlighted. **b)** Structural alignment of Pex1($_S$D2) (residues 868-1021, beige) and Pex6($_S$D2) (residues 900-1030, blue transparent). Note the distinct Pex1(α28) and Pex6(α38) protrusion domains highlighted by the dashed line. **c)** Contact site of Pex6($_S$D2) with Pex1($_L$D2) (compact Interface within the dimer). Hydrophobic residues (M951, L952, M955 and Y944) mediate interactions within the dimer binding into a hydrophobic groove (right panel) formed by Pex1(α18) and the loop upstream. **d)** Contact site of Pex1($_S$D2) with Pex6($_L$D2) (loose interface between dimers). Two hydrophobic residues (Y918 and Y921) on Pex1(α27) mediate interactions between dimers via binding into a hydrophobic pocket (right panel) formed by Pex6(α28) and the loop upstream.

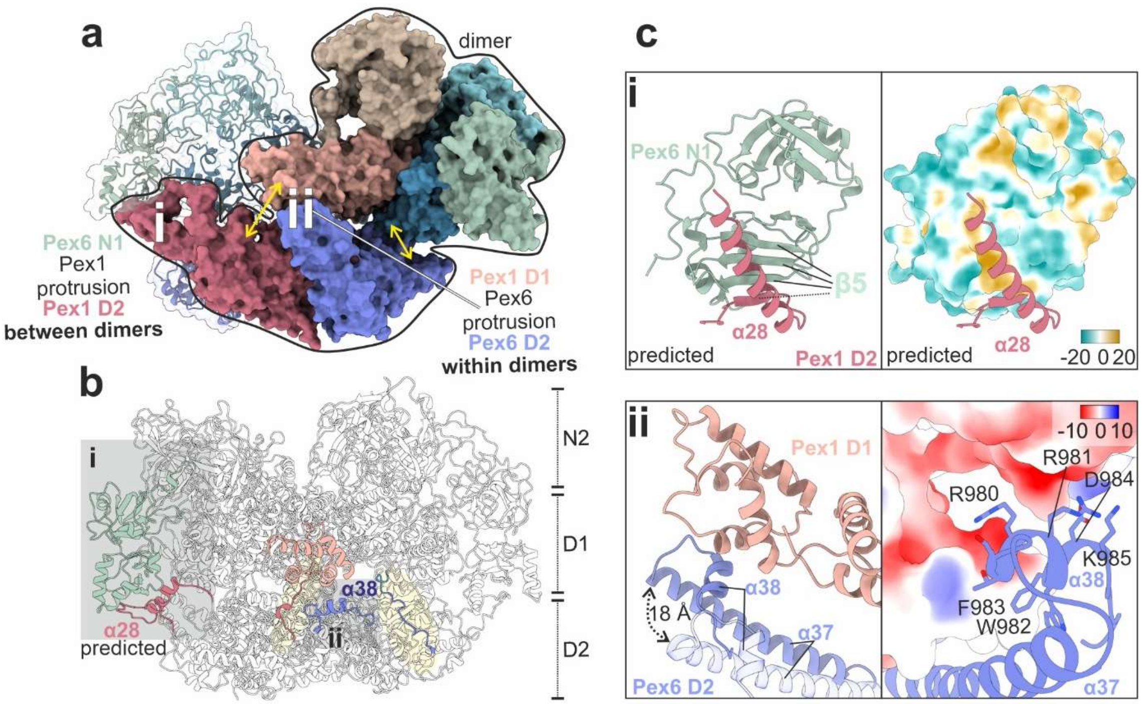


**Supplementary Figure 10: Interactions between the D1 and D2 ring. a)** Overview of the D1-D2 interactions via the protrusion domains of Pex1(i), Pex6(ii) and flexible linker peptides (yellow double-arrows). A Pex1/Pex6 subunit dimer is shown in surface representation. The clockwise (from top) adjacent Pex6 subunit is shown in ribbon representation. **b)** The structural elements involved in D1-D2 interactions are shown in ribbon representation and highlighted in color. In particular, the linker peptides (Pex1 (red, aa680-690); Pex6 (blue, residues 722-729)) covalently link the D1 and D2 ATPase cassettes. The protrusion domain of Pex1(D2) (amphipathic α28 helix) is flanked by long flexible loops and establishes an interface between neighboring dimers via anchoring at the adjacent Pex6(N1) domain (green) (i). The protrusion domain of Pex6(D2) (helix α38) mediates contact between the Pex6(D2) and the Pex1($_S$D1) domains within a subunit dimer (ii). **c)** Close-up view of interface (i) (upper-insets) and ii (lower insets). Please note: The Pex1(D2) protrusion domain (α28) is not well resolved. To address this, the interface (i) (Pex1(D2) (residues 940-976, red) and Pex6(N1)(1-201, green) (left panel) was predicted using Alphafold. Note that the Pex1 β-strand upstream of α28 enters the β-sheet 5 (β5) of Pex6. Together they form a hydrophobic groove, to which α28 (red) docks (right panel). **ii** This intra-dimer interface is flexible and depends on the nucleotide binding state of Pex6(D2). The Pex6(α38) protrusion helix at the tip of Pex6(α37) undergoes large movements (indicated by a dashed arrow) during ATPase cycle (right panel). During the transition from twin- to single seam state, α38 of the Pex6(D2) domain reengages and binds into a charged groove of Pex1(D1). Scalebar kcal/(mol·e) at 298 K.

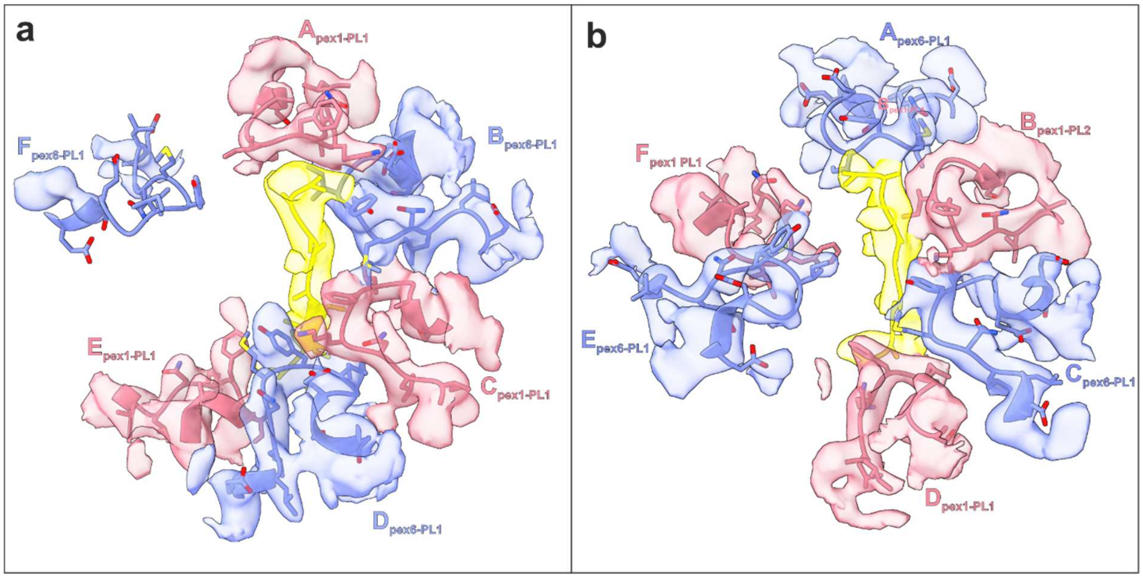


**Supplementary Figure 11: Density and molecular model of PL1 and substrate of single-seam (class3) (a) and twin-seam state (class4) (b).** Density-modified map is shown at 2.26 sigma (a, class3) and 2.11 sigma (b, class4).

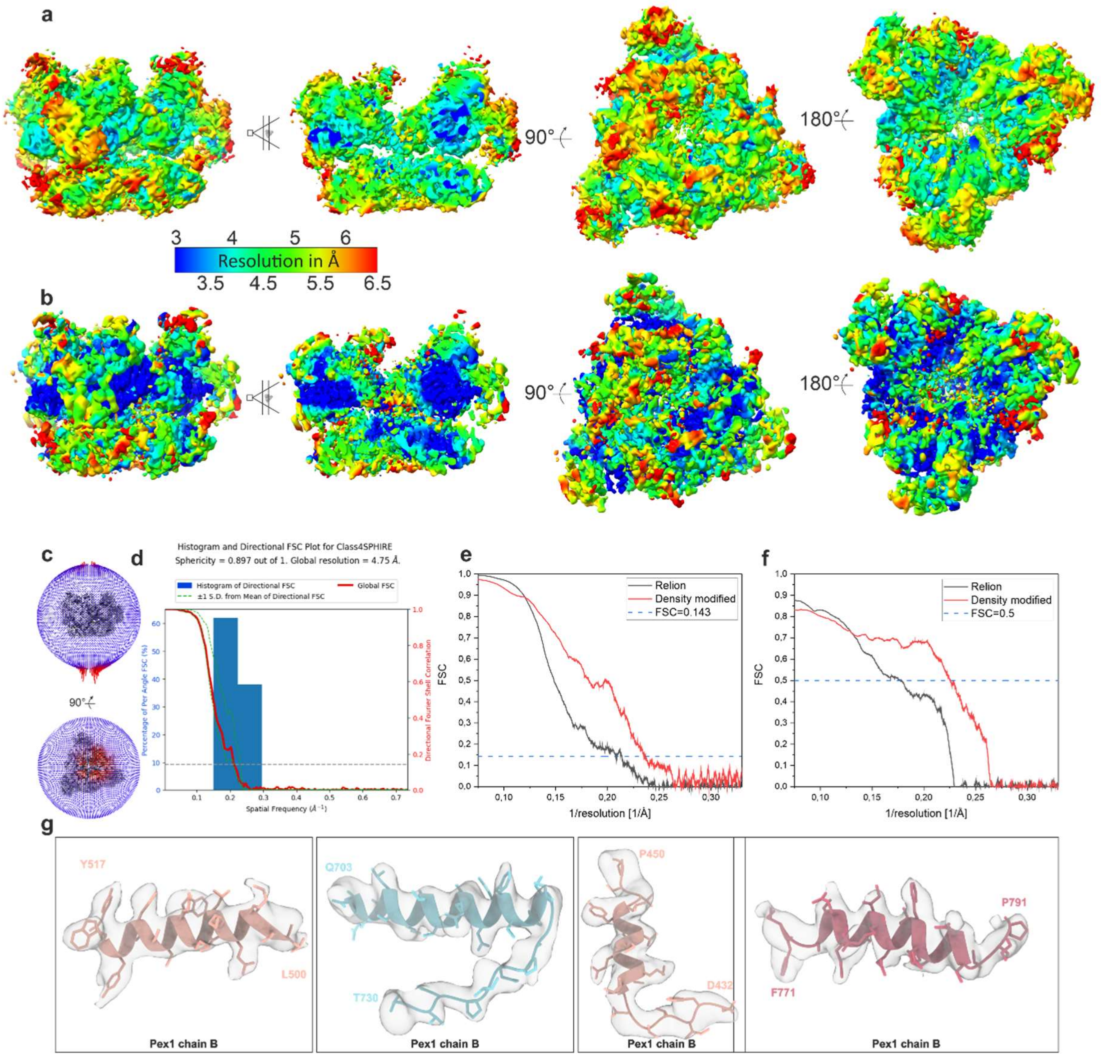


**Supplementary Figure 12: Cryo-EM structure of the single 'twin-seam' state (class 4). a-b)** Different surface views and cross-section of the cryo-EM density map colored according to the local resolution, upon post-processing in RELION at 4.42 sigma (a) and after local anisotropic half map sharpening and density modification using Phenix at 2.11 sigma (b). **c)** Angular distribution for the final round of the refinement. **d)** 3D FSC curve. **e)** Half-map FSC curves and **f)** model-map FSC curves before (gray) and after (red) density modification using phenix. **g)** Representative areas of the density map superimposed with the molecular model. Density-modified map is shown at 2.11 sigma.

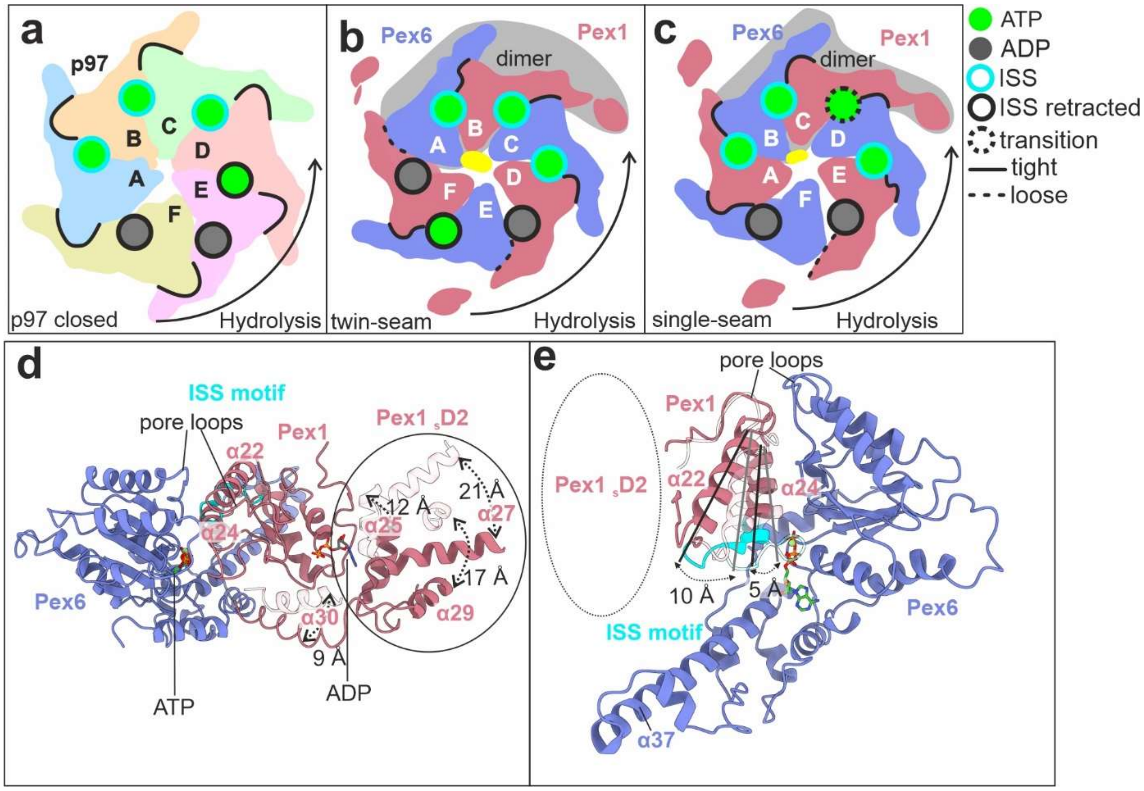


**Supplementary Figure 13: Inter-subunit signaling (ISS) motif controls ATP hydrolysis. a)** Illustration showing the arrangement of the D2 ring in p97 (closed conformation PDB: 7LN5)[2]. Note that the ISS motif of subunit F (ADP) is retracted from the nucleotide site of the neighboring subunit E and primes this site for hydrolysis. **b-c)** Illustration showing the arrangement of the D2 ring in Pex1/Pex6 in “single-seam” (b) and “twin-seam” state (c). Note that the ISS at the tight dimeric interface (D/E) (“single-seam”, b) blocks hydrolysis. Upon release, the dimer (E/F) (“twin-seam”, c) is less compact, the ISS is retracted and the dimeric interface is primed for hydrolysis. **d)** Pex6/Pex1 “twin-seam dimer” (chain E/F, colored blue/red) superimposed with staircase engaged Pex6/Pex1 dimer from the single-seam state (chain D/E, chain D removed, chain E transparent red) aligned on the large ATPase subdomain of Pex6 to visualize conformational changes upon dimer-detachment from the staircase. Note large conformational changes of the Pex1($_S$D2) subdomain upon detachment from the staircase. **e)** Same as in (**d**) but viewed from top. Pex1($_L$D2) is removing the ISS motif from the nucleotide pocket to prime it for hydrolysis.

**Source data**

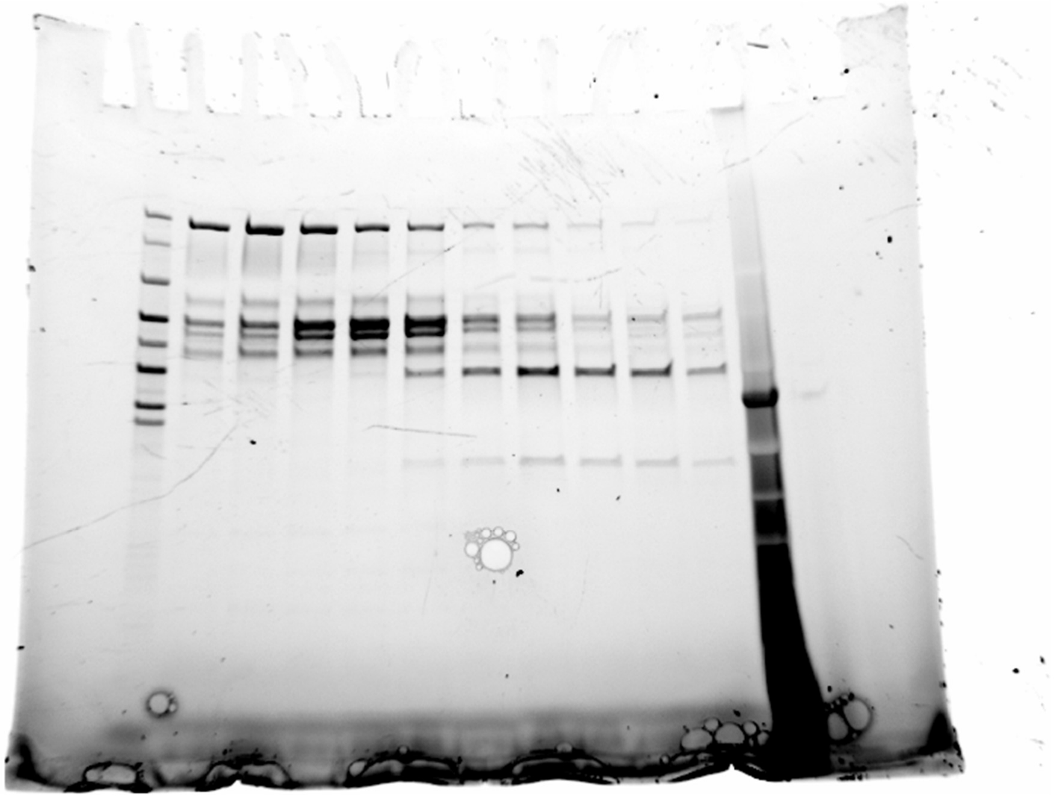

**Source data 1: Uncropped gel corresponding to Supplementary Figure 2a.**

# Supplementary References


1. Laskowski, R. A. PDBsum: summaries and analyses of PDB structures. *Nucleic acids research* **29,** 221–222; 10.1093/nar/29.1.221 (2001).

2. Pan, M. *et al.* Mechanistic insight into substrate processing and allosteric inhibition of human p97. *Nature structural & molecular biology* **28,** 614–625; 10.1038/s41594-021-00617-2 (2021).
