# Supplementary material for: Structure of the peroxisomal Pex1/Pex6 ATPase complex bound to a substrate
Source: Nat Commun. 2023 Sep 23;14:5942. doi: 10.1038/s41467-023-41640-9 (PMC10518020; doi:10.1038/s41467-023-41640-9)
Supplement: Supplementary file 2 — Reporting Summary [file 41467_2023_41640_MOESM2_ESM.pdf]

nature research

Corresponding author(s): Christos Gatsogiannis

Last updated by author(s): 23.08.2023

Reporting Summary

Nature Research wishes to improve the reproducibility of the work that we publish. This form provides structure for consistency and transparency in reporting. For further information on Nature Research policies, see our Editorial Policies and the Editorial Policy Checklist.

Statistics

For all statistical analyses, confirm that the following items are present in the figure legend, table legend, main text, or Methods section.

n/a	Confirmed	
☐	☒	The exact sample size (*n*) for each experimental group/condition, given as a discrete number and unit of measurement
☐	☒	A statement on whether measurements were taken from distinct samples or whether the same sample was measured repeatedly
☒	☐	The statistical test(s) used AND whether they are one- or two-sided<br>*Only common tests should be described solely by name; describe more complex techniques in the Methods section.*
☒	☐	A description of all covariates tested
☒	☐	A description of any assumptions or corrections, such as tests of normality and adjustment for multiple comparisons
☐	☒	A full description of the statistical parameters including central tendency (e.g. means) or other basic estimates (e.g. regression coefficient) AND variation (e.g. standard deviation) or associated estimates of uncertainty (e.g. confidence intervals)
☒	☐	For null hypothesis testing, the test statistic (e.g. *F*, *t*, *r*) with confidence intervals, effect sizes, degrees of freedom and *P* value noted<br>*Give P values as exact values whenever suitable.*
☒	☐	For Bayesian analysis, information on the choice of priors and Markov chain Monte Carlo settings
☒	☐	For hierarchical and complex designs, identification of the appropriate level for tests and full reporting of outcomes
☒	☐	Estimates of effect sizes (e.g. Cohen's *d*, Pearson's *r*), indicating how they were calculated

Our web collection on statistics for biologists contains articles on many of the points above.

Software and code

Policy information about availability of computer code

Data collection	EPU
Data analysis	TranSPHIRE ver.10 July 2020, SPHIRE ver 1.4, MotionCor2 ver.1.3.2, CTFFIND4 ver.4.1.14, crYOLO ver. 1.8.1, ISAC, RVIPER, MERIDIEN, RELION ver 3.1, PHENIX ver.1.20, WinCOOT ver.0.9.8, UCSF ChimeraX ver.1.4 to ver. 1.6, ISOLDE ver.1.4 to ver. 1.6, Chimera ver.1.15, iMODFIT, Alphafold 2 ver. 2.1.1, Corel Draw 2020, PyMol ver. 2.0.7, SnapGene ver.6.0.6, Proteome Discover 2.5

For manuscripts utilizing custom algorithms or software that are central to the research but not yet described in published literature, software must be made available to editors and reviewers. We strongly encourage code deposition in a community repository (e.g. GitHub). See the Nature Research guidelines for submitting code & software for further information.

Data

Policy information about availability of data

All manuscripts must include a data availability statement. This statement should provide the following information, where applicable:

- Accession codes, unique identifiers, or web links for publicly available datasets
- A list of figures that have associated raw data
- A description of any restrictions on data availability

Cryo-EM maps were deposited in the Electron Microscopy Data Bank (EMDB ID: 16372, 16373). Coordinates were deposited in the Protein Data Bank (PDB ID 8C0V, 8C0W) and are available upon publication. The raw cryo-electron movies and particle coordinates have been deposited deposited to EMPIAR (EMPIAR-11671) and are available upon publication. Mass-spectrometry data have been deposited to PRIDE and are available via ProteomeXchange with identifier PXD043907 upon publication.

Field-specific reporting

Please select the one below that is the best fit for your research. If you are not sure, read the appropriate sections before making your selection.

☒ Life sciences ☐ Behavioural & social sciences ☐ Ecological, evolutionary & environmental sciences

For a reference copy of the document with all sections, see nature.com/documents/nr-reporting-summary-flat.pdf

Life sciences study design

All studies must disclose on these points even when the disclosure is negative.

Sample size	16763 cryo-EM movies were collected using the EPU software, which was sufficient to obtain high-resolution cryo-EM volumes. This dataset is sufficient for high-resolution cryoEM image analysis as shown throughout the manuscript and the resulting maps. Representative cryoEM images are shown in Supplementary figure 2b and 2c.
Data exclusions	Low-quality data were excluded during single particle image processing in order to reach high-resolution using statistical methods. The exclusion criteria are well established as implemented in SPHIRE and Relion, which is a common practice in the cryo-EM field.
Replication	Protein purifications were performed several times on different days. For EM experiments, several negative stain EM and cryoEM grids were prepared. We selected the highest quality grid for data acquisition. Mass spectrometry and ATPase assay experiments were performed on different days. All replication attempts were successful.
Randomization	Randomization is not relevant to this structural study, because grouping is not required.
Blinding	Blinding is not relevant to this structural study; several crucial steps of the cryoEM processing workflow are automated.

Reporting for specific materials, systems and methods

We require information from authors about some types of materials, experimental systems and methods used in many studies. Here, indicate whether each material, system or method listed is relevant to your study. If you are not sure if a list item applies to your research, read the appropriate section before selecting a response.

Materials & experimental systems

n/a	Involved in the study	
☒	☐	Antibodies
☐	☒	Eukaryotic cell lines
☒	☐	Palaeontology and archaeology
☒	☐	Animals and other organisms
☒	☐	Human research participants
☒	☐	Clinical data
☒	☐	Dual use research of concern

Methods

n/a	Involved in the study	
☒	☐	ChIP-seq
☒	☐	Flow cytometry
☒	☐	MRI-based neuroimaging

Eukaryotic cell lines

Policy information about cell lines

Cell line source(s)	Saccharomyces Cerevisiae MH272/3fα wild type strain
Authentication	The S.c. MH272/3fα wild type strain has been kindly provided by Dr. Alexander Belyy (Max-Planck Institute Dortmund, Germany).
Mycoplasma contamination	It has not been tested for mycoplasma
Commonly misidentified lines (See ICLAC register)	N/A
