# Supplementary material for: Structure of the peroxisomal Pex1/Pex6 ATPase complex bound to a substrate
Source: Nat Commun. 2023 Sep 23;14:5942. doi: 10.1038/s41467-023-41640-9 (PMC10518020; doi:10.1038/s41467-023-41640-9)
Supplement: Supplementary file 4 — Description of Additional Supplementary Files [file 41467_2023_41640_MOESM4_ESM.pdf]

Description of Additional Supplementary Files

File name: Supplementary Movie 1

Description: Cryo-EM structure of the Pex1/Pex6 (class 3; "single-seam" state). Color code as in Figure 1a.

File name: Supplementary Movie 2

Description: Structural comparison between "twin-seam" and "single-seam" state. Color code as in Figure 1a. Note that the transition from twin-seam to single-seam state in the D2 ring induces a swing-in of the Pex1(D1/N2)/Pex6(D1/N2/N1) domains in the D1 ring (see Fig. 4d-e).

File name: Supplementary Movie 3

Description: Structural comparison of the Pex1(D2)/Pex1(D1) interface between "twin-seam" and "single-seam" state. (see Fig. 4g).
